# Manual Labor Schools

**Published:** 1860-10

**Authors:** 


### MANUAL LABOR SCHOOLS.

THESE were the rage a quarter of a century ago, but one by one they gradually died out; and at this time there is perhaps not a single one in existence. It is very certain, however, that they may be successfully conducted to the end of placing a young man on the stage of life in health of body, thoroughly educated, and with a mind independent and self-reliant; qualities, by the way, which are inseparable from any high character. Mr. Rose, of New-York, lately bequeathed three hundred thousand dollars for the purchase of a farm on which to place destitute children, on the condition that an equal amount should be contributed by other parties. The Hon. Charles Cook, of Havannah, Schuyler Co., New-York, offers this contribution, provided the farm is located at that place where there is a "PEOPLE'S COLLEGE" established, to which, by the munificence of this last-named gentleman, a farm is already attached, and shops are to be erected in which the students can work a few hours every day, the profits of their labor being placed to their credit.

The best and truest benevolence is to put a man in the way of helping himself; this gives him self-reliance: relieves him of the degradation of dependence, and makes him at once feel that he is a man—the highest aid and the best guarantee that he will act like a man. The very moment a youth becomes the recipient of a gratuity, the very first time the fact breaks in upon him that he has asked and received from one upon whom he has no claims, that he is a beggar, the *prestige* of manliness is gone, and he is ruined for life, as for all great purposes.

It is earnestly hoped that the "PEOPLE'S COLLEGE," at Havannah, will be a success, and that it will be the honored pioneer of a similar establishment in every State in the Confederacy. Some of the principles by which such institutions should be conducted, are as follows: They should be arranged so that a proficiency in scholarship, agriculture, and the more remunerative mechanical arts should be attainable, so that any young man may secure a collegiate education or become a proficient in farming or in some handicraft by which he may sustain himself when he goes out into the world to hew his way to fame or fortune.

A liberal price should be paid for the time spent in work, or for the job done. Such a price only as the article would command elsewhere. It is a great injury done to the receiver to pay him more for a thing than it is worth; it does an injury to society; there should be a just *quid pro quo*, no more, no less.

The morning should be devoted to study, the afternoon to labor, the night to sleep, to all that the body will receive. The brain will always work best in the morning; it is then most vigorous; works with greater ease and to better purpose; then bodily labor will be a recreation, a relief, a renovation. If the first part of the day is devoted to labor, the mind always will take up study with diminished alacrity.

The work and the study should be performed mainly in the same clothing; changing the garments loses time, and imposes a daily liability to taking cold, and endangers the health.

All should be required to be ready for study at sunrise throughout the year; to take breakfast at seven, dinner at one, and to retire uniformly at nine, winter and summer. The nights of winter should be devoted to lectures and questionings on physiology and hygiene, with other subjects which do not involve the special employment of the eyesight by artificial light. We have known Southern slaves to purchase themselves in a few years, by laboring at odd hours and Saturday afternoons, on a piece of land or in some mechanical calling at which they were adepts; and certainly young men could, with the work of half of each day in the year, pay all their expenses and leave the institution, owing no man any thing, and have several hundred dollars to start with—money of their own honest earnings—and be assured that, with such a start in life, the chances that such a young man would be heard of, sooner or later, in a sense honorable to himself and useful to society, is largely greater than if he had been left a fortune.

